# Photorespiration Alleviates Photoinhibition of Photosystem I under Fluctuating Light in Tomato

**DOI:** 10.3390/plants11020195

**Published:** 2022-01-12

**Authors:** Qi Shi, Hu Sun, Stefan Timm, Shibao Zhang, Wei Huang

**Affiliations:** 1 Kunming Institute of Botany, Chinese Academy of Sciences, Kunming 650201, China; shiqi@mail.kib.ac.cn (Q.S.); sunhu19@mails.ucas.ac.cn (H.S.); sbzhang@mail.kib.ac.cn (S.Z.); 2 University of Chinese Academy of Sciences, Beijing 100049, China; 3 Plant Physiology Department, University of Rostock, D-18051 Rostock, Germany; stefan.timm@uni-rostock.de

**Keywords:** photorespiration, cyclic electron flow, photoinhibition, photoprotection, photosystem I

## Abstract

Fluctuating light (FL) is a typical natural light stress that can cause photodamage to photosystem I (PSI). However, the effect of growth light on FL-induced PSI photoinhibition remains controversial. Plants grown under high light enhance photorespiration to sustain photosynthesis, but the contribution of photorespiration to PSI photoprotection under FL is largely unknown. In this study, we examined the photosynthetic performance under FL in tomato (*Lycopersicon esculentum*) plants grown under high light (HL-plants) and moderate light (ML-plants). After an abrupt increase in illumination, the over-reduction of PSI was lowered in HL-plants, resulting in a lower FL-induced PSI photoinhibition. HL-plants displayed higher capacities for CO_2_ fixation and photorespiration than ML-plants. Within the first 60 s after transition from low to high light, PSII electron transport was much higher in HL-plants, but the gross CO_2_ assimilation rate showed no significant difference between them. Therefore, upon a sudden increase in illumination, the difference in PSII electron transport between HL- and ML-plants was not attributed to the Calvin–Benson cycle but was caused by the change in photorespiration. These results indicated that the higher photorespiration in HL-plants enhanced the PSI electron sink downstream under FL, which mitigated the over-reduction of PSI and thus alleviated PSI photoinhibition under FL. Taking together, we here for the first time propose that photorespiration acts as a safety valve for PSI photoprotection under FL.

## 1. Introduction

Growth light significantly affects photosynthetic performance in plants. Plants usually modulate their biochemical composition and leaf morphology to acclimate to the specific growth light conditions [1,2,3,4,5]. In general, plants grown under high light (HL-plants) have higher content of proteins and enzymes involving in photosynthetic electron flow and the Calvin–Benson cycle than plants grown under low light [6,7]. These characteristics favors the higher photosynthetic capacity in HL-plants. Concomitantly, the rate of ribulose-1,5-bisphosphate (RuBP) oxygenation is also increased in HL-plants due to the higher ribulose-1,5-bisphosphate carboxylase/oxygenase (RubisCO) content [8]. Photorespiration is essential for the normal photosynthesis ambient CO_2_ and oxygen [9]. A stronger electron flow for photorespiration can protect photosystem II (PSII) by consuming the excess light energy [9,10]. However, the role of photorespiration in protecting photosystem I (PSI) under fluctuating light is not well known.

In natural habitats, leaves usually experience fluctuations of illumination owing to cloud, wind and changing leaf sun angle [11,12]. Under fluctuating light (FL), light absorption and PSII electron flow rapidly increased after an abrupt increase in light intensity [13,14]. Meanwhile, stomatal opening and the activation of the Calvin–Benson cycle have much slower kinetics [15,16,17]. Under such conditions, the reducing power in PSI cannot be immediately consumed by CO_2_ fixation. The resulting over-reduction of PSI triggers the donation of electrons to O_2_, producing reactive oxygen species in PSI [12,18,19]. Moreover, the antioxidant systems cannot immediately scavenge the reactive oxygen species [18]. Therefore, FL can give rise to PSI photoinhibition in many angiosperms [20,21,22,23,24]. Once PSI was damaged, CO_2_ assimilation and photoprotection were depressed, impairing the growth of plants [19,25,26,27,28].

To protect PSI under FL, plants have evolved several photoprotective strategies to optimize the PSI redox state [29,30,31,32]. In non-angiosperm plants, flavodiiron proteins mediate the photo-reduction of O_2_ and prevent PSI photoinhibition under FL, which is supplemented by cyclic electron flow (CEF) around PSI [29,32,33,34,35]. In angiosperms, the genes of flavodiiron proteins are lost and CEF is reserved to protect PSI under FL [19,36,37,38]. When light intensity increased abruptly, CEF activity rapidly increased to help the building up of trans-thylakoid proton gradient (ΔpH) [13]. Such CEF-dependent ΔpH formation can down-regulate the oxidation of plastoquinone and thus controls electron flow to PSI at the cytochrome (Cyt) b6/f complex [39]. Furthermore, the CEF stimulation can provide additional ATP, facilitating the operation of the primary metabolism [40]. Consequently, CEF significantly alleviates PSI photoinhibition under FL at donor and acceptor side [21]. In previous studies, plants grown under low light were usually used to investigate the role of CEF in PSI photoprotection under FL, and found that the donor side regulation was the primary target of CEF [19,20,21,38]. However, the underlying mechanism of acceptor side regulation in dependence of CEF have not yet been clarified. Furthermore, how HL-plants protects PSI under FL is poorly understood.

By transitioning from low to high light, the full activation of the Calvin–Benson cycle requires several minutes [41]. Meanwhile, photorespiration has relatively faster kinetics, making photorespiration to be a major alternative sink [42]. During photorespiration, the oxygenation of RuBP consumes high amounts of NADPH, leading to an increase in NADP^+^/NADPH ratio, which facilities the electron transport from PSI to NADP^+^ [43]. Under low CO_2_ concentration, the suppression of photorespiration by decreasing RubisCO content induced PSI over-reduction, and, thus accelerated PSI photoinhibition under excess light energy [44]. Therefore, photorespiration has the potential to promote the oxidation of PSI under excess light energy. The highly oxidation of PSI suppressed the donation of electrons from PSI to O_2_, and, thus prevented oxidative damage to PSI [45]. Accordingly, photorespiration might alleviate PSI photoinhibition under FL. We hypothesize that the increased capacity of photorespiration in HL-plants favors PSI photoprotection under FL.

In the present study, we measured gas exchange, chlorophyll fluorescence, P700 and electrochromic shift signals under fluctuating light for tomato plants grown under high light (HL-plants) and moderate light (ML-plants). Our aims were: (1) to compare the photosynthetic regulation under FL between HL- and ML-plants; (2) to assess the role of photorespiration in PSI photoprotection under FL. We found that FL induced a stronger PSI photoinhibition in ML-plants than HL-plants, and the higher capacity of photorespiration in HL-plants significantly alleviated FL-induced PSI photoinhibition.

## 2. Materials and Methods

### 2.1. Plant Materials

Tomato (*Lycopersicon esculentum* Miller cv. Hupishizi) plants were cultivated in full sunlight (HL-plants) or 40% full sunlight (ML-plants). The day/night air temperatures were approximately 30/20 °C, and the maximum light intensity at noon for HL- and ML-plants were approximately 2000 and 800 μmol photons m^−2^ s^−1^, respectively. All plants were grown with humus soil and any water and nutrient stress was prevented. After cultivation for one month, we used the canopy mature leaves for measurements.

### 2.2. Measurements of Gas Exchange and Photorespiration

We used an open gas exchange system (LI-6400XT; Li-Cor Biosciences, Lincoln, NE, USA) to simultaneously measure gas exchange and chlorophyll fluorescence. After photosynthetic induction at 1500 μmol photons m^−2^ s^−1^ and 400 μmol mol^−1^ CO_2_ concentration for 30 min, the net CO_2_ assimilation rates (*A*_N_) and chlorophyll fluorescence were recorded. The effective quantum yield of PSII photochemistry (Φ_PSII_) was calculated as (*F_m_*′ − *F_s_*)/*F_m_*′ [46]. The total electron transport rate through PSII (*J*_PSII_) as follows [47]:

(1) $$J_{\mathrm{PSII}}=\Phi_{\mathrm{PSII}}\times \mathrm{PPFD}\times 0.84\times 0.5$$

The electron transport rate for photorespiration was calculated as follows [48]:

(2) $$J_{O}=2/3\times \left(J_{\mathrm{PSII}}-4(A_{N}+R_{d}\right)$$

where *R*_d_ was measured after incubation in darkness for 20 min.

After adequate photosynthetic induction, the response of CO_2_ assimilation rate to incident intercellular CO_2_ concentration (*A*/*C*_i_) curves were measured by decreasing the CO_2_ concentration to a lower limit of 50 μmol mol^−1^ and then increasing stepwise to an upper limit of 1500 μmol mol^−1^. For each CO_2_ concentration, photosynthetic measurement was completed in 3 min. Using the *A*/*C*_i_ curves, the maximum rates of electron flux (*J*_max_) and RuBP carboxylation (*V*_cmax_) were calculated [49].

### 2.3. PSI and PSII Measurements

We used a Dual-PAM 100 measuring system (Heinz Walz, Effeltrich, German) to measure PSI and PSII parameters under atmospheric CO_2_ condition. After illumination at 1455 μmol photons m^−2^ s^−1^ for 5 min to activate photosynthetic electron sinks, leaves were exposed to FL alternating between low light (59 μmol photons m^−2^ s^−1^, 2 min) and high light (1455 μmol photons m^−2^ s^−1^, 1 min). PSI parameters were calculated as follows: Y(I) = (*P*_m_’ − *P*)/*P*_m_; Y(ND) = *P*/*P*_m_; Y(NA) = (*P*_m_ − *P*_m_′)/*P*_m_. Y(I), the quantum yield of PSI photochemistry; Y(ND), the quantum yield of PSI non-photochemical energy dissipation due to donor side limitation; Y(NA), the quantum yield of PSI non-photochemical energy dissipation due to acceptor side limitation. PSII parameters were calculated as follows: Y(II) = (*F_m_*′ − *F_s_*)/*F_m_*′; NPQ = (*F_m_* − *F_m_*′)/*F_m_*′; Y(NO) = *F_s_*/*F_m_*. Y(II), the effective quantum yield of PSII photochemistry; NPQ, non-photochemical quenching in PSII; Y(NO), the quantum yield of non-regulatory energy dissipation in PSII. The relative photosynthetic electron transport rate through PSI and PSII were calculated as: rETRI = PPFD × Y(I) × 0.84 × 0.5; rETRII = PPFD × Y(II) × 0.84 × 0.5. rETRI minus rETRII is assumed to be the rate of CEF.

### 2.4. Electrochromic Shift Measurement

A Dual-PAM 100 equipped with a P515/535 emitter-detector module (Heinz Walz) was used measure the electrochromic shift (ECS) signals. After light adaptation at 1455 μmol photons m^−2^ s^−1^ for 5 min, leaves were illuminated at 59 μmol photons m^−2^ s^−1^ for 2 min. Afterwards, light intensity was changed to 1455 μmol photons m^−2^ s^−1^, and ECS dark interval relaxation kinetics (DIRK_ECS_) were recorded after this light transition for 10 s or 60 s. The proton gradient (ΔpH) component of proton motive force were calculated using DIRK_ECS_ [50,51]. The chloroplast ATP synthase activity (*g*_H_^+^) was estimated as the inverse of the decay time constant of the first-order ECS relaxation [52].

### 2.5. Statistical Analysis

We determined whether significant differences existed between HL- and ML-plants using *t*-test (*α* = 0.05). The software SigmaPlot 10.0 was used for graphing and fitting.

## 3. Results

**Figure 1:**
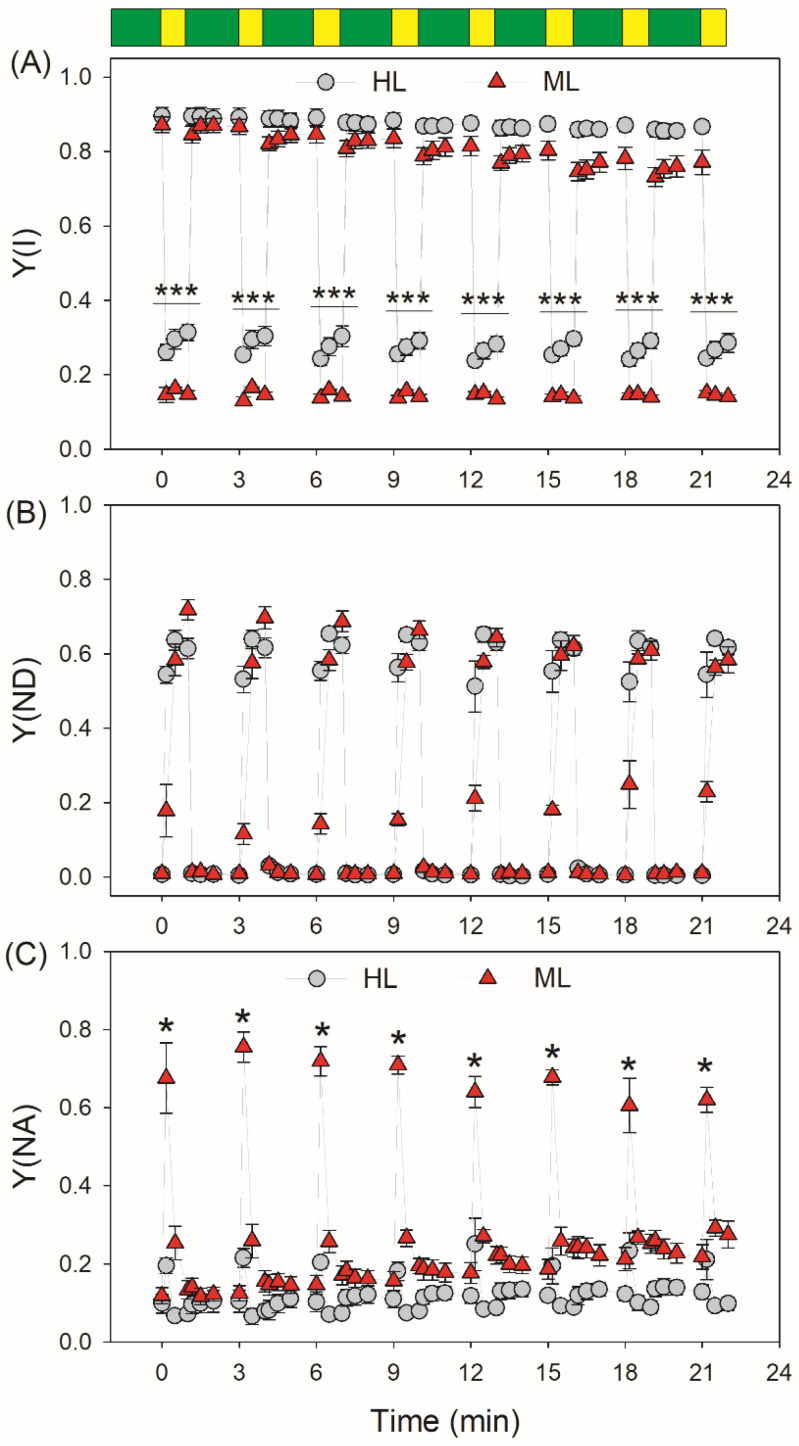
Changes in PSI parameters during fluctuating light in HL- and ML-plants of tomato. (**A**) Y(I), the quantum yield of PSI photochemistry; (**B**) Y(ND), the quantum yield of PSI non-photochemical energy dissipation due to the donor side limitation; (**C**) Y(NA), the quantum yield of PSI non-photochemical energy dissipation due to the acceptor side limitation. Data are shown as means ± SE (n = 5). Green bars indicate low light (59 μmol photons m^−2^ s^−1^); yellow bars indicate high light (1455 μmol photons m^−2^ s^−1^). Asterisk indicates a significant difference between HL- and ML-plants.

**Figure 2:**
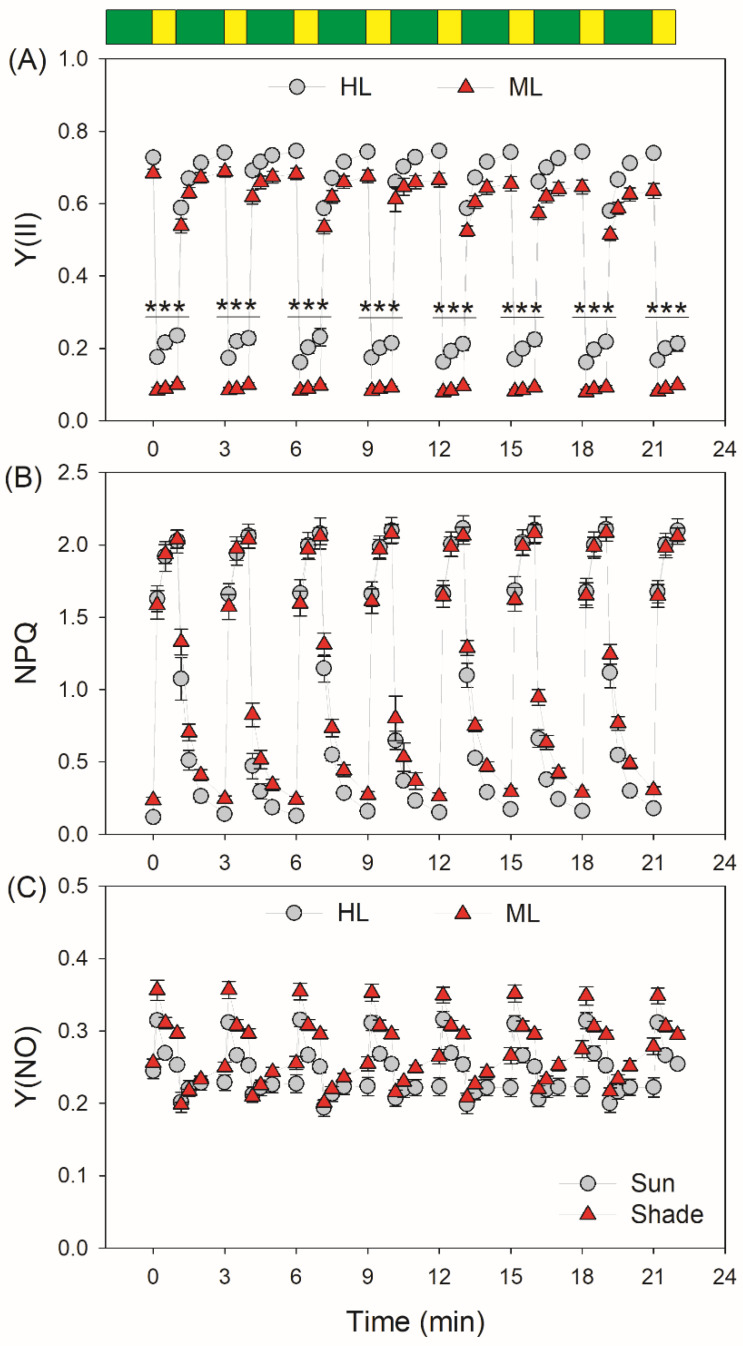
Changes in PSII parameters during fluctuating light in HL- and ML-plants of tomato. (**A**) Y(II), the effective quantum yield of PSII photochemistry; (**B**) NPQ, non-photochemical quenching in PSII; (**C**) Y(NO), the quantum yield of non-regulatory energy dissipation in PSII. Data are shown as means ± SE (n = 5). Green bars indicate low light (59 μmol photons m^−2^ s^−1^); yellow bars indicate high light (1455 μmol photons m^−2^ s^−1^). Asterisk indicates a significant difference between HL- and ML-plants.

Upon the light intensity changing from LL (59 μmol photons m^−2^ s^−1^) to HL (1455 μmol photons m^−2^ s^−1^), the quantum yield of PSI photochemistry (Y(I)) rapidly decreased in both types of leaves (Figure 1A). Under high-light phases, HL-plants had higher Y(I) values than ML-plants (Figure 1A). At LL, the quantum yield of energy dissipation due to donor-side limitation, Y(ND), was similar in HL- and ML-plants. However, after the transition from LL to HL for 10 s, Y(ND) increased more quickly in HL-plants (Figure 1B). Concomitantly, the quantum yield of energy dissipation due to acceptor-side limitation, Y(NA), increased to a much higher level in ML-plants (Figure 1C), indicating that the PSI over-reduction under FL was aggravated in ML-plants. Similar to the performance of Y(I), the effective quantum yield of PSII photochemistry, Y(II), rapidly decreased in both types of leaves by transitioning from LL to HL (Figure 2A). Furthermore, HL-plants displayed higher Y(II) values under high-light phases (Figure 2A). After transition from LL to HL for 10 s, non-photochemical quenching in PSII, NPQ, rapidly increased to approximately 80% of the maximum level in both HL- and ML-plants (Figure 2B), diminishing the quantum yield of non-regulatory energy dissipation in PSII (Y(NO)) (Figure 2C). Furthermore, ML-plants had higher Y(NO) than HL-plants, indicating more excess light energy in ML-plants.

**Figure 3:**
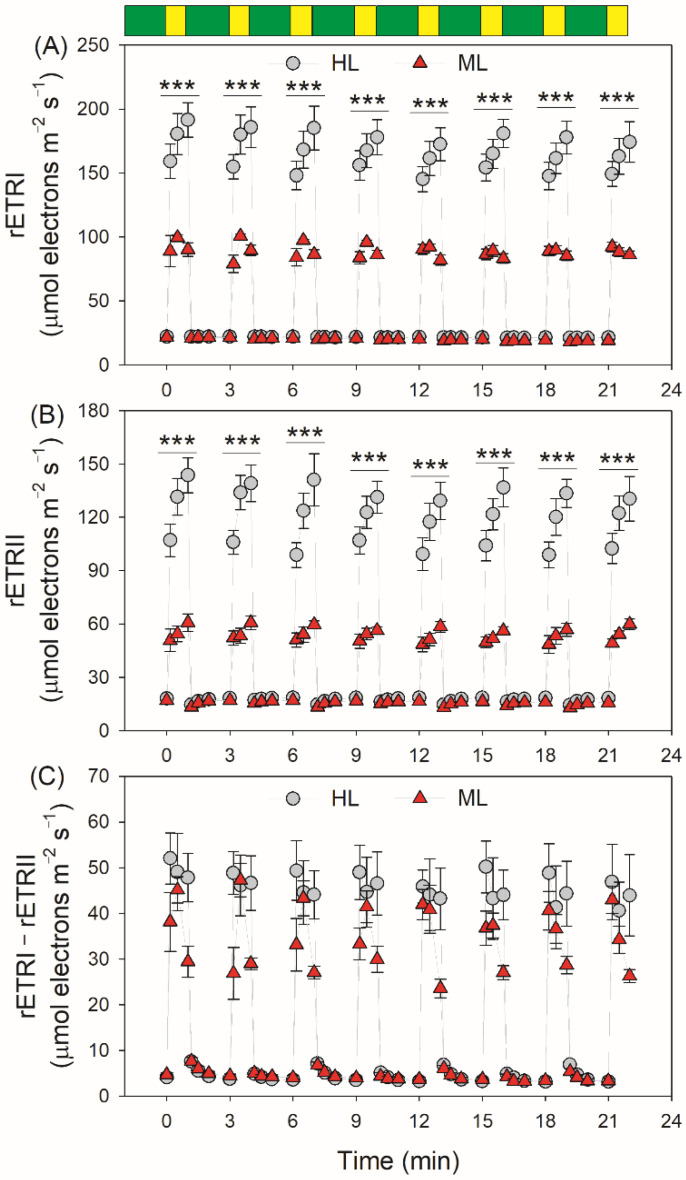
Changes in relative photosynthetic electron transport rates (rETRs) during fluctuating light in HL- and ML-plants of tomato. (**A**) rETRI, ETR through photosystem PSI; (**B**) rERTII, ETR through PSII; (**C**) rETRI–rETRII, estimated rate of cyclic electron flow. Data are shown as means ± SD (n = 5). Green bars indicate low light (59 μmol photons m^−2^ s^−1^); yellow bars indicate high light (1455 μmol photons m^−2^ s^−1^). Asterisk indicates a significant difference between HL- and ML-plants.

By transitioning from LL to HL, the relative PSI electron flow (rETRI) progressively increased in HL-plants (Figure 3A). By comparison, rETRI first increased and then decreased in ML-plants. The relative PSII electron flow (rETRII) progressively increased in both types of leaves, and HL-plants showed much higher rETRI and rETRII values in high-light phases (Figure 3B). In HL-plants, the rETRII values after this light transition for 60 s were much higher than those for 10 s. By comparison, rETRII just increased slightly in ML-plants. These results indicated that, within the initial 10 s after this light transition, the CO_2_ assimilation was strongly restricted in HL-plants. In order to evaluate the performance of CEF under fluctuating light, we analyzed the change in rETRI–rETRII during FL treatment. HL- and ML-plants showed similarly low values of rETRI–rETRII under LL. Interestingly, the changing pattern of CEF under FL varied between HL- and ML-plants (Figure 3C). After the transition from LL to HL, rETRI–rETRII rapidly increased to the maximum value in 10 s and maintained stable over time in HL-plants. By comparison, rETRI–rETRII firstly increased and subsequently rapidly decreased in ML-plants. In the first four cycles of LL/HL, the increase of CEF to the maximum value in ML-plants needed 30 s, indicating the delayed CEF activation in ML-plants. After the abrupt increase in illumination, the rETRI/rETRII ratio first increased and then decreased, suggesting the transient CEF stimulation under FL (Figure S1). Furthermore, the contribution of CEF to total photosynthetic electron flow was enhanced in ML-plants (Figure S1).

**Figure 4:**
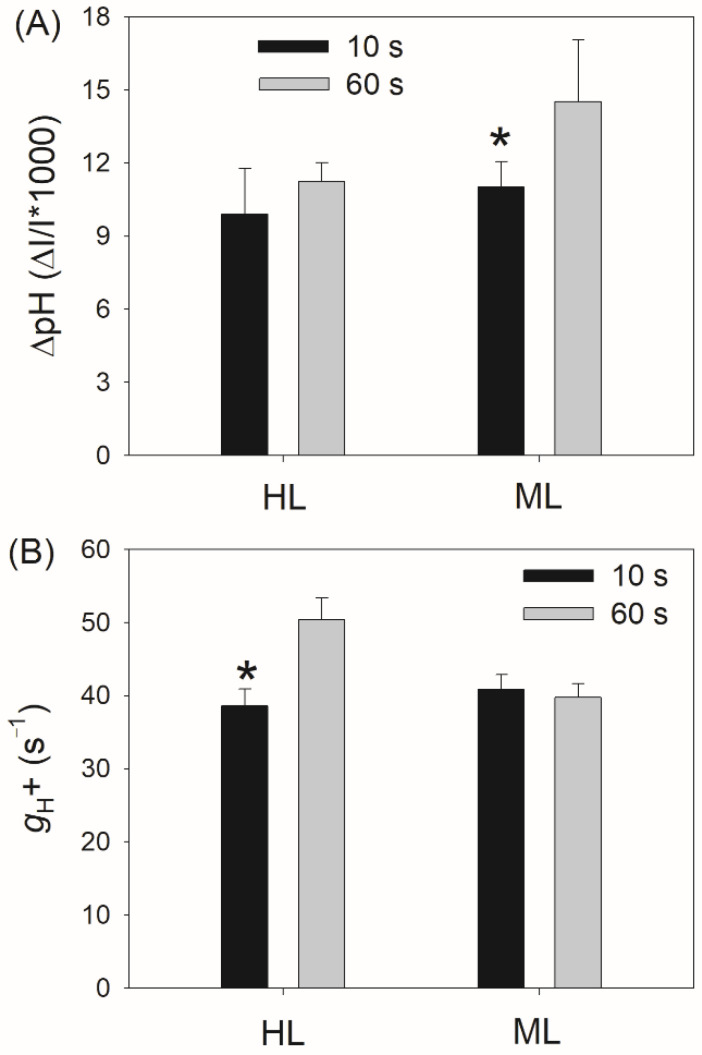
Changes in proton gradient (ΔpH) (**A**) and chloroplast ATP synthase activity (*g*_H_^+^) (**B**) during fluctuating light in HL- and ML-plants of tomato. ΔpH and *g*_H_^+^ were measured after transition from 59 to 1455 μmol photons m^−2^ s^−1^ for 10 s and 60 s. Data are shown as means ± SE (n = 5). Asterisk indicates a significant difference between 10 s and 60 s.

Because ΔpH significantly regulates PSI redox state and NPQ induction under high light [53,54], we next examined the change in ΔpH under fluctuating light. After transition from LL to HL for 10 s, the ΔpH value was slightly lower than that for 60 s in HL-plants (Figure 4A), suggesting that the ΔpH formation within the first 10 s was almost sufficient for photosynthetic regulation in HL-plants. By comparison, the ΔpH value for 10 s was significantly lower than that for 60 s in ML-plants (Figure 4A), indicating that the ΔpH formation within the first 10 s was insufficient to regulate photosynthetic apparatus in ML-plants. After this light transition, the chloroplast ATP synthase activity (*g*_H_^+^) gradually increased in HL-plants but was maintained stable in ML-plants (Figure 4B). Because a decrease in *g*_H_^+^ can enhance the formation of ΔpH, the flexibility of *g*_H_^+^ upon a sudden transition from LL to HL in HL-plants likely favored the rapid generation of ΔpH.

**Figure 5:**
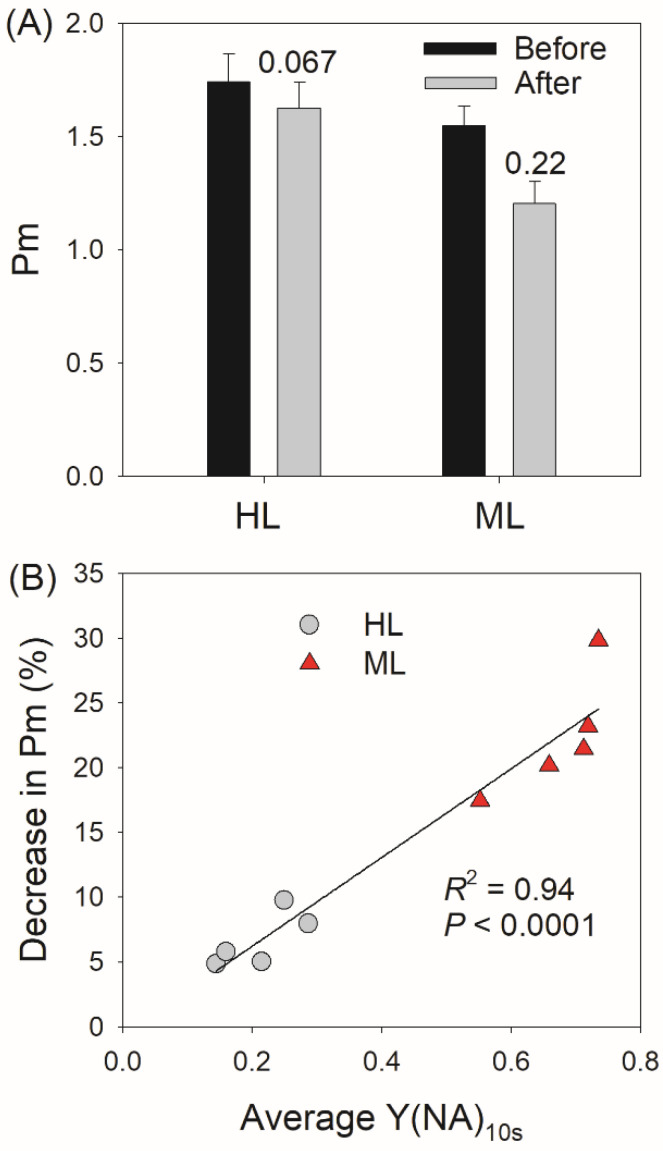
(**A**) The decrease in Pm after fluctuating light treatment for 24 min in HL- and ML-plants of tomato, and the decreasing amplitude was displayed in it. (**B**) Relationships between the decrease in Pm and the average Y(NA) after transition from 59 to 1455 μmol photons m^−2^ s^−1^ for 10 s during fluctuating light treatment in HL- and ML-plants. Data are shown as means ± SE (n = 5). Asterisk indicates a significant difference between HL- and ML-plants. Each symbol represents an individual leaf.

After fluctuating light treatment for eight cycles of low/high light, ML-plants showed a larger decrease in Pm than HL-plants (Figure 5A). Thus, FL induced a stronger PSI photoinhibition in ML-plants. Furthermore, we found a tight positive relationship between PSI photoinhibition and Y(NA) after transition from LL to HL for 10 s (Y(NA)_10s_) (Figure 5B). Therefore, the greater PSI photoinhibition in ML-plants induced by FL was mainly caused by the transient PSI over-reduction.

**Figure 6:**
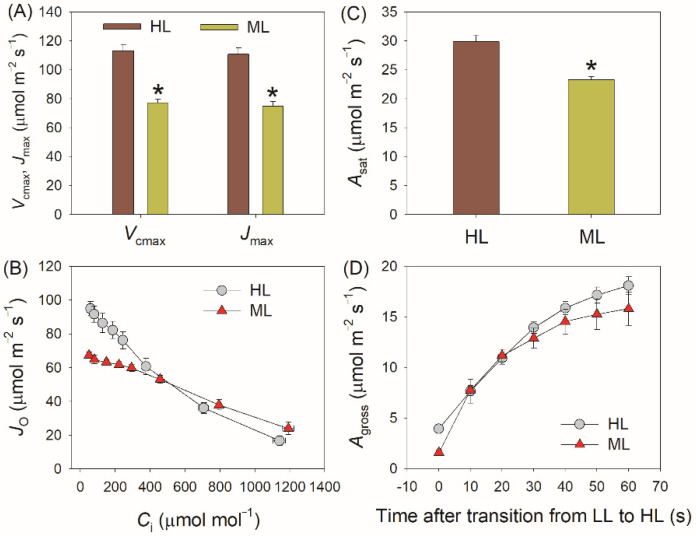
(**A**) The maximum rates of RuBP carboxylation (*V*_cmax_) and regeneration (*J*_max_), (**B**) response of electron flow to photorespiration (*J*_O_) to incident intercellular CO_2_ concentration (*C*_i_), (**C**) the saturating net CO_2_ assimilation rate (*A*_sat_) at 1500 μmol photons m^−2^ s^−1^ and 400 μmol CO_2_ mol^−1^, and (**D**) change in gross CO_2_ assimilation rate (*A*_gross_) after transition from 50 to 1500 μmol photons m^−2^ s^−1^ in HL- and ML-plants. Data are shown as means ± SD (n = 5). Asterisk indicates a significant difference between HL- and ML-plants.

**Figure 7:**
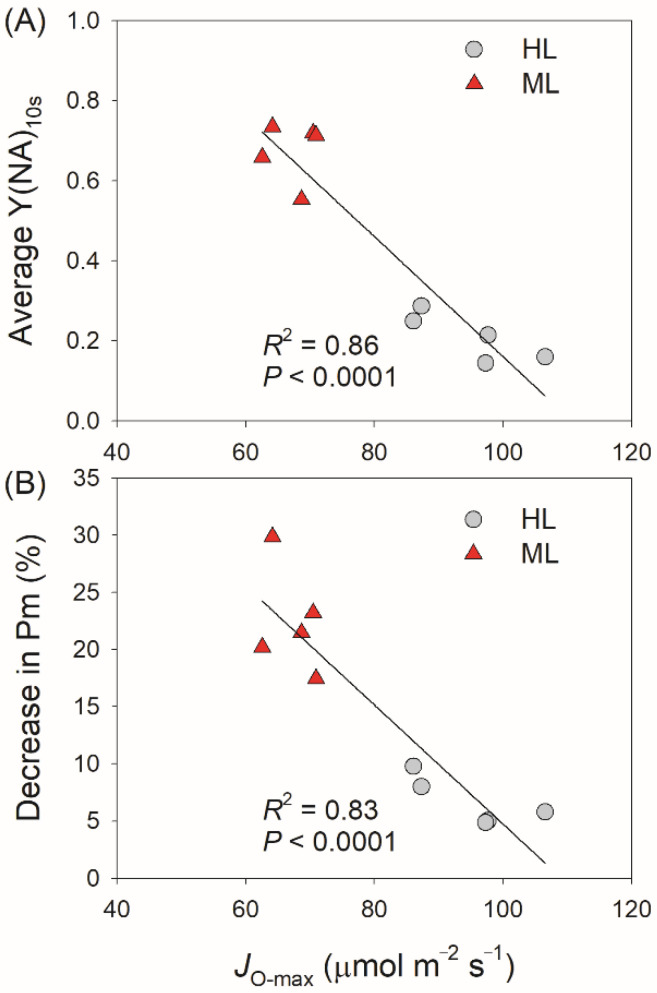
Effects of the maximum electron flow to photorespiration (*J*_O-max_) on the average Y(NA) after transition from 59 to 1455 μmol photons m^−2^ s^−1^ for 10 s (**A**) and the decrease in Pm (**B**) during fluctuating light treatment in HL- and ML-plants. *J*_O-max_ was obtained from the CO_2_ response curves in Figure 6.

The PSI redox state under FL is significantly affected by the electron sink downstream of PSI [38,55,56]. Compared with ML-plants, HL-plants not only displayed higher capacities for the maximum rates of RuBP carboxylation (*V*_cmax_) and regeneration (*J*_max_), and photosynthetic CO_2_ assimilation rate (*A*_sat_), but also showed higher electron flow for photorespiration (*J*_O_) when CO_2_ assimilation was restricted at low CO_2_ concentrations (Figure 6). Therefore, the electron sink downstream of PSI was significantly enhanced in HL-plants. Within the first 60 s after transition from 50 to 1500 μmol photons m^−2^ s^−1^, the gross CO_2_ assimilation rate did not differ between HL- and ML-plants (Figure 6), indicating that the variation of Y(NA)_10s_ between HL- and ML-plants was caused by photorespiration rather than the Calvin–Benson cycle. Furthermore, we found the maximum *J*_O_ was negatively correlated to the PSI over-reduction under FL and FL-induced PSI photoinhibition (Figure 7), indicating that the enhancement of photorespiration in HL-plants alleviated the PSI over-reduction under FL and thus mitigated PSI photoinhibition.

## 4. Discussion

After an abrupt increase in illumination, the slow kinetics of CO_2_ assimilation led to the lack of NADP^+^ and made PSI to be over-reduced owing to the restriction of electron flow from PSI to NADP^+^. Some previous studies reported that FL induced a marked PSI photoinhibition in some angiosperms [20,21,57,58]. Furthermore, in *Arabidopsis thaliana* (Arabidopsis) and *Erigeron annuus*, FL-induced PSI photoinhibition was stronger in LL-plants than in HL-plants [59]. Therefore, growth light can significantly affect the response of PSI to FL. However, the underlying mechanisms are not clear. Here we demonstrated that, upon transition from LL to HL, tomato ML-plants significantly showed a stronger PSI over-reduction than HL-plants, ultimately causing higher PSI photoinhibition in ML-plants. Therefore, ML-plants can fine-turn the redox state of PSI under FL to a much lesser extent. Plants grown under high light might display slight damage of PSI FeS cluster after exposure to high light [60]. In this study, we measured all photosynthetic parameters in the morning (a.m. 9:00–12:00), which might exclude the possibility of PSI photodamage in HL-plants.

The PSI redox state under FL is largely affected by donor and acceptor side regulation [21]. During donor side regulation, a high ΔpH can down-regulates the plastoquinone (PQ) oxidation at the Cyt *b*_6_/*f* complex, which restricts the electron transport from PQ to plastocyanin (PC) and, thus, avoids excess electron flow to PSI [61]. If ΔpH formation under high light was impaired, PSI would be over-reduced, leading to uncontrolled PSI photoinhibition [62,63]. Therefore, a sufficient ΔpH is indispensable to PSI photoprotection under high light. The ΔpH formation under high light is mainly affected by photosynthetic electron flow and chloroplast ATP synthase activity [53,64]. After transition from LL to HL, HL-plants displayed high levels of photosynthetic electron transport rates and a relatively low chloroplast ATP synthase activity, contributing to the sufficient ΔpH formation. By comparison, the ML-plants generated an insufficient ΔpH after this light transition for 10 s. Therefore, HL-plants reinforced the PSI donor side regulation under FL.

In PSI acceptor side regulation, reducing power in PSI is consumed by linear electron flow, photo-reduction of O_2_ mediated by the Mehler reactions and Flvs [33,65,66]. Flv-dependent alternative electron flow was lost in angiosperms during evolution [37]. The O_2_ photo-reduction mediated by the Mehler reactions, called water-water cycle, could rapidly consume excess electrons in PSI and thus avoided PSI over-reduction under FL [56,67,68]. In the present study, a transient PSI over-reduction under FL indicated that water-water cycle was not significant in tomato leaves. The excess PSII electron flow is the prerequisite for PSI over-reduction [36,63,69,70]. Although HL-plants displayed much higher PSII electron flow than ML-plants, HL-plants showed a rapid oxidation of PSI upon the sudden transition from LL to HL. These results suggested that HL-plants have the ability to rapidly consume the large amounts of reducing power in PSI through acceptor side regulation.

After transition from LL to HL for 10 s, the value of rETRII was much higher in HL-plants than ML-plants (Figure 3B), whereas the gross CO_2_ assimilation rate showed no significant difference between them (Figure 6). These results indicated that, upon a sudden increase in illumination, the difference in rETRII between HL- and ML-plants was not attributed to the Calvin–Benson cycle but was caused by the change in photorespiration. Accordingly, within the first 10 s after this light transition, electron flow for photorespiration was the major component of PSII electron flow in HL-plants. Furthermore, photorespiration could rapidly consume the excess reducing power in PSI [42]. An increase in photorespiratory pathway can accelerate the consumption of NADPH and subsequently elevates the NADP^+^/NADPH ratio. Therefore, the enhanced photorespiratory pathway in HL-plants facilitates the outflow of electrons from PSI to NADP^+^ under FL and eventually alleviates PSI over-reduction at acceptor side. Furthermore, the higher electron flow to photorespiration in HL-plants helped the rapid formation of ΔpH after transition from low to high light, which alleviated PSI over-reduction under FL at donor side. Taking together, the enhancement of photorespiration in HL-plants protected PSI against photoinhibition under FL at donor and acceptor sides.

The photorespiratory pathway starts with the oxygenation of RuBP, which leads to the formation of 3-phosphoglycerate (3-PGA) and 2-phosphoglycolate (2-PG) [71]. 2PG is a dead-end intermediate of photosynthesis, which cannot directly be used and strongly impairs plant carbon metabolism [72,73,74]. To prevent the accumulation of 2-PG, photorespiratory pathway converts 2-PG into 3-PGA to replenish the Calvin–Benson cycle [75,76]. Therefore, acceleration of the photorespiratory pathway can significantly boost CO_2_ assimilation rate and plant growth via optimized RuBP regeneration and preventing 2-PG mediated down-regulation of the Calvin–Benson cycle [43,77,78,79]. Thus, the enhancement of photorespiratory pathway has the potential to improve plant growth and crop yield under field FL conditions. Under FL conditions, avoiding PSI photoinhibition is a premise of the maintenance of CO_2_ assimilation rate [31,80]. This is in good agreement with the presented results and the hypothesis that that the photorespiratory pathway acted as a safety valve to protect PSI under FL.

In previous studies on PSI photoprotection under FL, CEF is thought to be the main factor affecting the PSI redox state. During CEF, electrons are transferred from ferredoxin to PSI, which is accompanied with the proton translocation [81,82,83]. The CEF-dependent formation of ΔpH is critical for NPQ induction and PSII photoprotection under high light [84]. Moreover, once CEF activity was decreased by mutation of PGR5- and/or NDH-pathway, the PSI over-reduction under high light would be aggravated, leading to severe PSI photoinhibition [62,63,80,85]. After transition from LL to HL, CEF first increased and then rapidly decreased in ML-plants, which was consistent with previous results reported in *Arabidopsis* and tobacco plants grown under low light [20,22]. The first transient CEF stimulation helped the formation of ΔpH. The latter decrease in CEF prevented an over-acidification of thylakoid lumen, which maximized the photosynthetic light use efficiency. However, we found that the CEF performance in HL-plants was different from ML-plants. After the same light transition, the rate of CEF rapidly increased and then was maintained stable in HL-plants. Furthermore, within the first 30 s after transition from LL to HL, ML-plants displayed higher rETRI/rETRII ratios than HL-plants (Figure S1). These results indicated that ML-plants showed a larger contribution of CEF to total photosynthetic electron transport under FL. Therefore, to prevent uncontrolled over-reduction of PSI under FL, ML-plants enhanced the stimulation of CEF to compensate for the shortage of photorespiration.

## 5. Conclusions

We here showed that ML-plants displayed a greater PSI over-reduction under FL than HL-plants in tomato. As a result, FL induced a stronger PSI photoinhibition in ML-plants. Furthermore, the up-regulation of photorespiration in HL-plants enhanced the outflow of electrons from PSI under FL and thus alleviated PSI photoinhibition. Therefore, photorespiration acts as an important valve for PSI protection under FL. In ML-plants, CEF was highly activated to compensate for the lower capacity of photorespiration, which prevented uncontrolled PSI photoinhibition. Taking together, HL- and ML-plants likely use different strategies to protect their photosynthetic apparatus under FL. Future studies involving defined photorespiration mutants are needed to shed more light on these aspects.

## Data Availability

The data presented in this study are available on request from the corresponding author.

## Supplementary Materials

The following supporting information can be downloaded at: https://www.mdpi.com/article/10.3390/plants11020195/s1, Figure S1: Changes in the rETR/rETRII ratio during fluctuating light in HL- and ML-plants of tomato. Green bars indicate low light (59 μmol photons m^−2^ s^−1^); yellow bars indicate high light (1455 μmol photons m^−2^ s^−1^). Data are shown as means ± SE (n = 5).
