# Supplementary material for: Photorespiration Alleviates Photoinhibition of Photosystem I under Fluctuating Light in Tomato
Source: Plants (Basel). 2022 Jan 12;11(2):195. doi: 10.3390/plants11020195 (PMC8780929; doi:10.3390/plants11020195)
Supplement: Supplementary file 1 [file plants-11-00195-s001.zip › plants-1517646 supplementary.pdf]

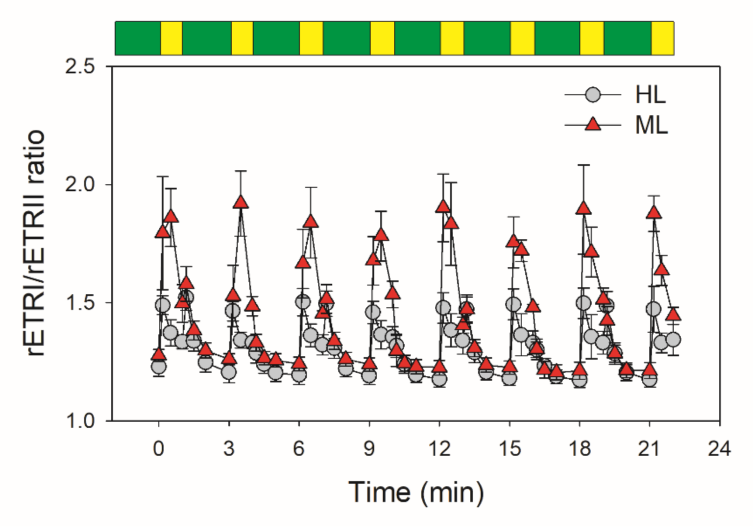

Figure S1. Changes in the rETR/rETRII ratio during fluctuating light in HL- and ML-plants of tomato. Green bars indicate low light (59 μmol photons m^{-2} s^{-1}); yellow bars indicate high light (1455 μmol photons m^{-2} s^{-1}). Data are shown as means ± SE (n = 5).
